# Microfluidics-Based Single-Cell Research for Intercellular Interaction

**DOI:** 10.3389/fcell.2021.680307

**Published:** 2021-08-12

**Authors:** Long Pang, Jing Ding, Xi-Xian Liu, Zhixuan Kou, Lulu Guo, Xi Xu, Shih-Kang Fan

**Affiliations:** ^1^School of Basic Medical Science, The Shaanxi Key Laboratory of Brain Disorders, Xi’an Medical University, Xi’an, China; ^2^Xi’an Key Laboratory of Pathogenic Microorganism and Tumor Immunity, Xi’an Medical University, Xi’an, China; ^3^Department of Mechanical and Nuclear Engineering, Kansas State University, Manhattan, KS, United States; ^4^Key Laboratory of Thermo-Fluid Science and Engineering of MOE, School of Energy and Power Engineering, Xi’an Jiaotong University, Xi’an, China

**Keywords:** cell-cell communication, cell culture, single-cell manipulation, single-cell analysis, microfluidic technology

## Abstract

Intercellular interaction between cell–cell and cell–ECM is critical to numerous biology and medical studies, such as stem cell differentiation, immunotherapy and tissue engineering. Traditional methods employed for delving into intercellular interaction are limited by expensive equipment and sophisticated procedures. Microfluidics technique is considered as one of the powerful measures capable of precisely capturing and manipulating cells and achieving low reagent consumption and high throughput with decidedly integrated functional components. Over the past few years, microfluidics-based systems for intercellular interaction study at a single-cell level have become frequently adopted. This review focuses on microfluidic single-cell studies for intercellular interaction in a 2D or 3D environment with a variety of cell manipulating techniques and applications. The challenges to be overcome are highlighted.

## Introduction

Intercellular interaction, including cell–cell and cell–ECM communication, is pivotal to multicellular organisms. Communication errors can cause diseases like cancer metastasis, motor neuron disease, virus–host interaction, and diabetes (Schwager et al., 2019; Toda et al., 2019; Gromova and Spada, 2020; Reyes-Ruiz et al., 2020). For this reason, the intercellular interaction study can improve the understanding of pathogenic mechanism and advance drug development. However, challenges remain in the analysis of the mechanisms of intercellular interaction, as impacted by the sophisticated intercellular interaction networks in microenvironments (You et al., 2002; Daneshpour and Youk, 2019).

Traditional methods to study intercellular interaction are to maintain the native microenvironment *in vivo*, which are commonly limited by the availability of expensive equipment and the complicated processes (Huang Q. et al., 2019; Pang et al., 2019b). A more effective method of intercellular interaction research is to employ *in vitro* tools that can significantly simplify the isolation and control of the microenvironment. Plenty of methods *in vitro* have been used for intercellular interaction studies. The Boyden chamber, which is also called the transwell chamber, consists of two compartments separated by a microporous membrane, has been used for intercellular intercation research, such as differentiation, secretion, and migration (Kaneda et al., 2019; Kumar et al., 2011). Because of the simplicity and stability, this method continues to be employed (Goers et al., 2014). The defects of the Boyden chamber lie in the lack of physiological relevance and the limit of spatial control. Moreover, the Boyden chamber assay is difficult to study a small amount of cells or single cells, and to integrate with downstream analyses (e.g., protein-protein interactions, RNA-Seq, and ChIP-Seq). Alternative systems include Petri dishes and co-culture in gels or bioreactors. The shortcomings of the traditional methods are low flexibility and low compatibility with other analysis processes (Vu et al., 2017).

Microfluidics-based systems for cell–cell and cell–ECM communication studies have recently become practical. The advantages of the microfluidics-based systems are low reagent consumption, precise reagent manipulation, high throughput, and easy integration of functional components (Sackmann et al., 2014; Huang Q. et al., 2019). The microfluidics-based system can delve into intercellular interaction both on a population basis and on a single-cell level. Over the past decades, microfluidics-based systems have been utilized to study intercellular interaction at population levels with demonstrated merits and demerits (Zervantonakis et al., 2011; Guo et al., 2013; Konry et al., 2016; Vu et al., 2017; Rothbauer et al., 2018). Recently, advanced microfluidics-based systems for cell–cell communication at a single-cell level have been adopted for biological and medical studies (Luo et al., 2019; Sakthivel et al., 2019). In contrast to a group of cells, single-cell microfluidics-based systems exhibit numerous advantages. For instance, as cells are heterogeneous and varied in numerous aspects like mechanical characterization and protein expression, microfluidics-based systems can isolate and study individual cells, including circulating tumor cells (CTCs) and stem cells (Gupta et al., 2010; Cheng et al., 2018; Pang et al., 2020). Intercellular interaction at a single-cell level is valuable in understanding communication pathways and commutating behaviors of special subpopulations of cells, which could be employed for the studies of secretion, differentiation, and migration (Lu et al., 2017; Alonso et al., 2019).

**FIGURE 1:**
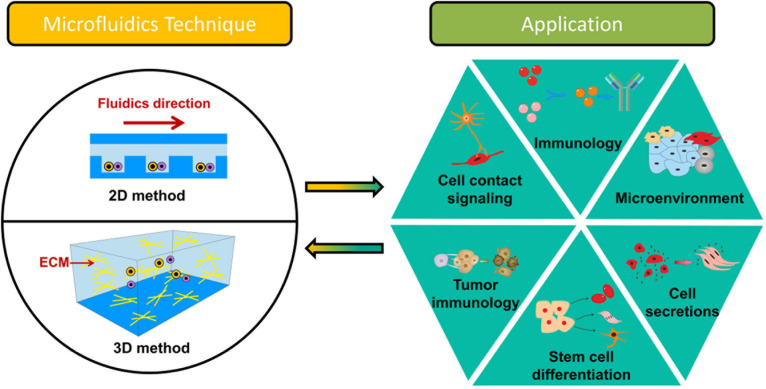
Overview of microfluidics-based systems for intercellular interaction study at the single-cell level.

Generally, based on the way that cells interact with each other, microfluidics-based systems for intercellular interaction studies at a single-cell level could be discussed based on 2D (two-dimensional) and 3D (three-dimensional) methods as shown in Figure 1. 2D microfluidics-based systems usually focus on the communication of homotypic or heterotypic cells at an identical surface (Li et al., 2019; Tavakoli et al., 2019). Although many materials [e.g., poly(methyl methacrylate), polystyrene, and fluorinated thermoplastic polymers] have been used for microfluidics-based systems for cell–cell communication studies, the most commonly method is based on polydimethylsiloxane (PDMS) devices fabricated by soft lithography. The advantages of using PDMS devices are easy fabrication and good permeability to gas (e.g., O_2_ and CO_2_), allowing complicated and long-time 2D cell–cell communication studies (Vu et al., 2017). Though the 2D methods are favored for simple quantification of gene expression, physiology and cell morphology, 3D microfluidics-based systems could study more complex interactions on different dimensions. 3D microfluidics-based systems are able to perform interactions between cell and cell and between cell and extracellular matrix (ECM) (Nahavandi et al., 2014; Lee et al., 2018; Ali et al., 2019). ECM, a surrounding of a complex molecular composition and fibers, creates structural support and thereby allows cells to grow three-dimensionally (Cukierman et al., 2002; Yamada and Cukierman, 2007). ECM mainly contains collagen, elastin, glycoproteins, and polysaccharides (Dutta and Dutta, 2009). In the past decades, many natural biomaterials [e.g., gelatin hydrogel (GA), hyaluronic acid (HA), and matrigel] have been used for 3D cell-culture *in vitro* (Li et al., 2019; Perebikovsky et al., 2021). GA is a subtype of collagen, which can be isolated from bones, ligaments, and tendons. GA could exhibit different mechanical properties due to the sources and extraction processes. Due to the low cost and low antigenicity, GA has been widely used in the biomedical field. HA, which is present in connective tissues, could be used for the studies of cell migration, proliferation and inflammatory diseases. Matrigel is derived from the basement membrane (BM) of the Engelbreth–Holm–Swarm (EHS) mouse sarcoma; It is often crosslinked with collagen for intercellular intercation study. Additionally, there are many (semi)synthetic-based hydrogels [e.g., polyethylene glycol (PEG), polylactic acid (PLA), or poly(lactic-co-glycolic acid) (PLGA)] used for modeling the ECM (Morales et al., 2021). Unlike native biomaterials, they do not exhibit functional ligands for cells and hence require crosslinking with native proteins or chemical insertion of matrix metalloproteinase (MMP)-sensitive peptides and integrin-binding domains [RGD (Arg-Gly-Asp) motifs]. In the present review, we categorize the microfluidic devices as 2D and 3D. Both 2D and 3D intercellar communication and their applications are demonstrated. The relationship between organ-on-a-chip and intercellular interactions at the single-cell level are described. Lastly, the challenges are addressed.

## 2D Microfluidic Systems

**FIGURE 2:**
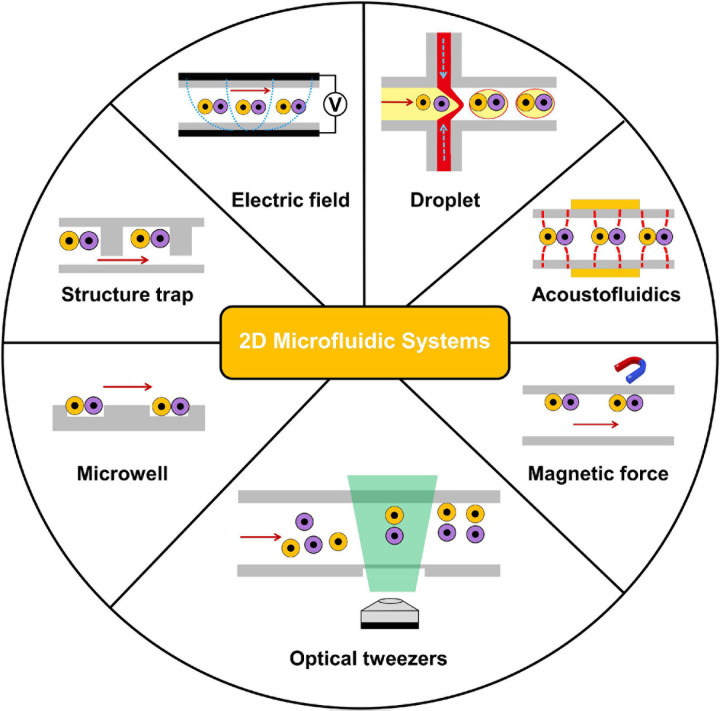
Overview of 2D microfluidics-based systems for intercellular interaction study at the single-cell level. 2D microfluidic systems are classified based on different cell positioning methods: microwell, structure trap, electric field, droplet, acoustofluidics, magnetic force, and optical tweezers.

In the past decades, 2D microfluidics-based systems for studies at a single-cell level have been extensively applied. With 2D microfluidic cell–cell communication systems, two cells could be spatially paired near each other to record their interactions (Klepárník and Foret, 2013; Mu et al., 2013). As the single-layer nature of numerous microfluidic devices, the 2D approach could be easier developed on a chip (Ertl et al., 2014; Huang J. et al., 2019). As shown in Figure 2, 2D microfluidic systems are classified based on different cell positioning methods: microwell, structure trap, electric field, droplet, acoustofluidics, magnetic force, and optical tweezers in this section. Table 1 compares the 2D approaches.

**TABLE 1:** Microfluidic devices and their applications in 2D cell–cell communication.

**Method**	**Throughput**	**Cell pairing rate**	**Application**	**Cell type**	**Reference**
Microwell	N/A	N/A	Heterotypic cell pair	Rat ventricular myocyte (NRVM) and human embryonic kidney 293 (HEK293) cell	McSpadden et al., 2012
	1,000–5,000 wells	25%	Heterotypic cell pair	Natural killer (NK) cell and K562 cell	Yamanaka et al., 2012
	N/A	70 %	Heterotypic cell pair	Kasumi-1 cell, NK-92 cell, CCRF-SB cell, and Ramos cell	Kim et al., 2019
	36,100 (190 × 190 array) wells	40%	Heterotypic cell pair	Rat primary hepatocyte and PC-3 prostate cancer cell	Lee et al., 2015
	6,400 wells	N/A	Heterotypic cell pair	Dynamic CD8^+^ T cells (isolated from OT-1 mouse) and murine acute myeloid leukemia cells (C1498)	Tu et al., 2020
Structure trap	150 traps	50%	Heterotypic cell pair	Mouse embryonic fibroblast (MEF) and mouse embryonic stem cell (mESC)	Hong et al., 2012
	6,000 traps	70%	Heterotypic cell pair and fusion	NIH/3T3 fibroblasts, myeloma cells, B cells, mouse embryonic stem cell (mESCs) and mouse embryonic fibroblasts (mEFs)	Skelley et al., 2009
	750–900 traps in ∼2 × 3 mm^2^	80%	Homotypic/Heterotypic cell pair and fusion	eGFP-expressing NIH/3T3, DsRed-expressing NIH/3T3 and BA/F3 mouse leukocyte	Dura et al., 2014
	648 cell-enclosing units	85%	Heterotypic cell pair	NK-92 cell and K562 human erythroleukemia cells	Li et al., 2017
	500–850 traps mm^–2^	40–85%	Heterotypic cell pair	Dynamic CD8^+^ T cells (from OT-1 mice) and SIINFEKL-loaded MHCII-eGFP B cells	Dura et al., 2015
	440 traps	N/A	Hematopoietic cell pair	Primary T cell and dendritic cell (DC)	Faley et al., 2008
	N/A	N/A	Hematopoietic cell pair	Normal CD34^+^ and CML CD34^+^ hematopoietic stem cells	Faley et al., 2009
	4,000 traps	>70%	Heterotypic cell pair	CD16–KHYG-1 cells (KHYG-1 human NK cell expressing CD16) and the K562 myelogenous leukemia cell	Jang et al., 2015
	200 traps	70%	Heterotypic and homotypic cell pair and co-culture	Human SW480 epithelial cell, HT29 colon carcinoma and MCF-7 epithelial-like breast cancer cells	Frimat et al., 2011
	N/A	71.1%	Homotypic cell pair and co-culture	HeLa cell	Lin et al., 2013
	N/A	94%	Heterotypic cell pair and co-culture	MDA-MB-231/GFP, MDA-MB-436/RFP, and MCF-7/GFP cells	Zhang et al., 2014
	N/A	67-100%	Homotypic cell pair and co-culture	Untreated MCF-7 cell and thapsigargin-treated MCF-7 cell	Fatsis-Kavalopoulos et al., 2019
	N/A	25%	Heterotypic cell pair and co-culture	UM-SCC-1 cell and endothelial cell	Chen et al., 2014a
	80 traps	84%	Heterotypic and homotypic cell pair	HUVEC (Human Umbilical Vein Endothelial Cell), HeLa cell and MCF-7 cell	Zhu et al., 2019
	N/A	>50%	Multiple cells pair and co-culture	Human Oral squamous cell carcinoma (OSCC) TW2.6 expressing WNT5B-specific shRNA, OSCC TW2.6 pLKO-GFP cell, and lymphatic endothelial cell (LECs)	He et al., 2019
	N/A	>50%	Multiple cells pair	HeLa cell, HT-29 cell, and NIH/3T3 fibroblast	Tang et al., 2020
	N/A	N/A	Particle pair	20.3 and 10.1 μm particles	Lee and Burns, 2015
	30 valve mm^–2^	92.1%	Particle pair	30 or 100 μm particles	Kim et al., 2017
	N/A	N/A	Homotypic cell pair	10 μm polystyrene beads, HEK cell	Duchamp et al., 2019
Electric field	N/A	20%	Heterotypic cell fusion	Jurkat, NG 108-15, PC-12, and Cos-7 cell	Strömberg et al., 2000
	384-well plate	N/A	Cell microenvironment	Immortalized human umbilical vein cells (iHUVEC)	Yin et al., 2010
	384-well plate	N/A	Homotypic cell pair and co-culture	K562 leukemia cells	Bocchi et al., 2012
	(>2,400 pairs) in a 1×1.5 cm^2^	74.2%	Homotypic cell pair	HeLa cell	Wu et al., 2017
	N/A	80%	Heterotypic cell pair and co-culture	Prostate cancer (PC-3) cell and myoblast (C2C12) cell	Chen et al., 2014b
Droplet	N/A	13%	Heterotypic cell pair and co-culture	Mating-type minus (mt-) and mating-type plus (mt+) *vegetative C. reinhardtii* cell	Lagus and Edd, 2013
	103 trapping sites	N/A	Homotypic cell pair	Primary T cell and dendritic cell (DC)	Sarkar et al., 2015
	N/A	N/A	Heterotypic cell pair	CD8^+^ T cell, dendritic cell (DC), RPMI-8226 cell [multiple myeloma (MM) cell]	Sarkar et al., 2016
	1152 trapping sites	88.1%	Particle pair	15 μm fluorescent particle and 30 μm non-fluorescent particle	Chung et al., 2017
	N/A	16.2%	Heterotypic cell pair	Jurkat E6.1 cell and K562 cell	Segaliny et al., 2018
	4,000 trapping sites	N/A	Heterotypic cell pair	CD8^+^ T cell, MDA-MB-231 cell and SKOV3 cells	Sullivan et al., 2020
Acousto-fluidics	N/A	73%	Heterotypic cell pair and co-culture	HEK 293T cell, hTERT-HMVEC (human microvascular endothelial cells, CRL-4205) and HeLa S3 (CCL-2.2) cell	Guo et al., 2015
	N/A	30%	Heterotypic cell pair cell with different size	*P. falciparum* parasite, red blood cell (RBC) and lymphocytes	Collins et al., 2015
Magnetic force	N/A	N/A	Heterotypic cell pair	B cell and T cell isolated from the spleens of C57BL6 mice	Lim et al., 2014
Optical tweezers	77 pairs	N/A	Homotypic cell pair	Normal and system lupus erythematosus (SLE) red blood cells (RBCs)	Khokhlova et al., 2012
	N/A	N/A	Homotypic cell pair	Human pluripotent stem cell (hPSC)	Jing et al., 2018
	N/A	N/A	Heterotypic cell pair	Human embryonic stem cell (hESC) and primary human dermal fibroblasts (HDFns)	Chen et al., 2013

### Microwell

**FIGURE 3:**
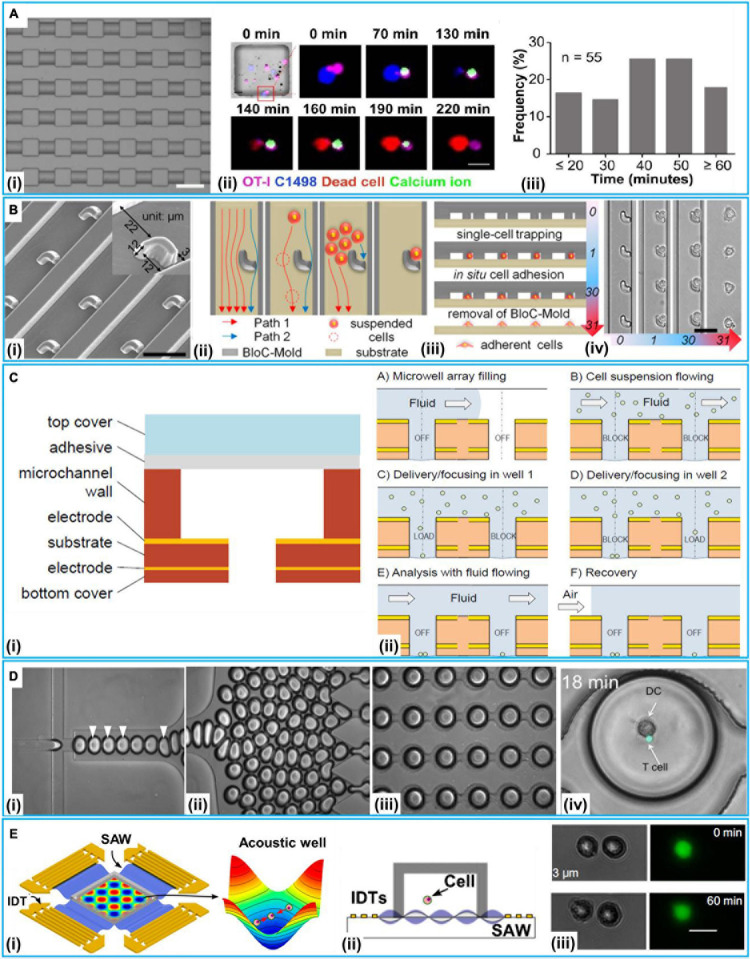
2D microfluidic systems at a single-cell level. **(A)** Microwell method. (i) Microscopic image. (ii) The real-time imaging of immunological synapse formation between T cells (OT-I) and leukemia cells (C1498) via calcium imaging. (iii) The frequency distribution of the time duration from Ca^2+^ entered into OT-I cells to leukemia cells death. Reproduced with permission from Tu et al. (2020), copyright 2020 The Royal Society of Chemistry. **(B)** The procedure of hook-shaped traps for cell pairing. (i) Scanning electron micrograph of the trap microarray. (ii) Schematic diagram of cell flow paths. Cross-sectional schematics (iii) and corresponding bright-field micrographs (iv) showing the entire BloC-Printing process. Reproduced with permission from Zhang et al. (2014), copyright 2014 The National Academy of Sciences of the United States. **(C)** The Electric-field-based open microwell system for cell pairing. (i) The build-up of the open microwell array. (ii) The procedure for cell pairing. Reproduced with permission from Bocchi et al. (2012), copyright 2012 The Royal Society of Chemistry. **(D)** Droplet method for co-encapsulation of a naive T cell and a DC-T cell. (i) Droplet generation at flow-focusing junction; droplets containing T cells indicated by arrowheads. (ii) Generated droplets driven toward the docking microarray. (iii) Droplet-filled microarray (iv) Co-encapsulation of naive T cell and DC. Reproduced with permission from Sarkar et al. (2015), copyright by the Sarkar et al. **(E)** Acoustofluidics for cell–cell interactions study. (i) Illustration of the experimental setup and the function of the acoustic well with proper pressure gradients and pressure nodes to concentrate cells. (ii) Schematic of experimental setup. (iii) Two HEK 293T cells positioned with a distance of 3 μm without dye transfer after 60 min, scale bar: 20 μm, Reproduced with permission from Guo et al. (2015), copyright 2014 The National Academy of Sciences of the United States.

A simplest method for 2D cell–cell communication systems is to adopt microwells. Microwells could control the number of cells by the size of each wells. With microwells, one can rely on the probability to capture a pair of desired cells together inside a well (Rettig and Folch, 2005; Hwang et al., 2012). McSpadden et al. (2012) reported a platform integrating microwell and microcontact printing methods to couple unexcitable donor cells with host cardiomyocytes under functional consequences. With such a platform, the pairing of a neonatal rat ventricular myocyte (NRVM) with an engineered human embryonic kidney 293 (HEK293) cell was carried out. Interactions of natural killer (NK) and cancer cells are critical to immunological control of cancer (Irimia and Wang, 2018). Yamanaka et al. (2012) employed arrays of sub-nanoliter wells (nano-wells) to monitor single NK cell–K562 cell [human immortalized myelogenous leukemia cell line, histocompatibility complex (MHC) class I-deficient] interactions. With this platform, the relationship between the secretion of interferon-γ (IFN-γ) from NK cell and target cell (K562) cytolysis was analyzed. Moreover, Kim et al. (2019) used microwells to accomplish an array of immobilized single hematological cancer cells; microwell size and surface coating were enhanced to maximize loading of single hematologic cells. On the demonstrated microwell array, quantitative study of lymphocyte cytotoxicity at the single-cell level was carried out with NK-92 cells against leukemic cells (CCRF-SB cells). Except for controlling the size of the microwells, Lee et al. (2015) presented an L-microwell for trapping single-cell in a respective branch via stretching/releasing of a PDMS substrate. The pair of single PC3 cancer cell and macrophage was obtained to monitor the diffusion of cell secreted molecules over 5,000 cell pairs on a 2.25 cm^2^ array. Recently, Tu et al. developed a device to establish a cell–cell interaction assay for profiling dynamic CD8^+^ T cells (isolated from OT-1 mouse) and murine acute myeloid leukemia cells (C1498) interactions at the single-cell level. This device could be used to test different cancer immunotherapy by comparing single T cells’ responses to different treatments (Figure 3A; Tu et al., 2020). This device was reported to hold great potential in testing clinical treatment for acute myeloid leukemia (e.g., CAR-T therapy and immune checkpoint blockade therapies). The heterogeneous cytotoxicity of T cells under immune checkpoint therapy was investigated, and the result confirmed that anti-PD1 (programmed cell death protein-1) had a positive influence on the cell killing ability of T cells.

### Structure Trap

Another frequently employed method of 2D microfluidics-based cell–cell communication systems is to exploit structure traps. The traps could capture the single-cell pairs based on cell size or/and deformability (Jin et al., 2014; Huber et al., 2018). Specifically, cells flowed along a path of least fluidic resistance to be trapped or bypass along the channels (Tan and Takeuchi, 2007). Once a trap was taken up by one cell, fluidic resistance increased, and the following cells would be directed to the next traps. Structure traps were extensively employed in stem cell, immune cell, and cancer cell microenvironment studies.

A simulated microenvironment *in vitro* is pervasive in stem cell studies. Hong et al. (2012) presented a platform for studies of dynamic cellular interactions between single mouse embryonic fibroblasts (MEF) and mouse embryonic stem cells (mESC). MEF and mESC pairs were performed via sequential cell trapping and dynamic variation of fluidic resistance. Cell fusion step is critical to initiate the stem cell reprogramming. Skelley et al. (2009) adopted a structure trap-based method to pair thousands of cells for cell fusion. Their device performed more than 50% properly paired and fused cells, which could observe reprogramming in hybrids between mESCs and MEF. Additionally, fused mESC-MEF hybrids could adopt an ESC-like morphology. The staining of alkaline phosphatase showed evidence of reprograming as judged by reactivation of endogenous Oct4-GFP reporters. Subsequently, they developed a method based on cell deformability with a fusion yield up to 95% with electro-fusion (Dura et al., 2014); NIH/3T3 fibroblasts and BA/F3 mouse leukocytes, eGFP- and DsRed-expressing NIH/3T3 mouse fibroblasts were paired, then eGFP- and DsRed-expressing NIH/3T3 mouse fibroblasts were fused based on various biological, chemical, and physical stimuli.

Moreover, many structure trap methods have been applied to the observation of immune cells. Li et al. (2017) demonstrated a microfluidic cell loading-dock system (Cell-Dock) which presented precise and dynamic cell packings to study immune cell cytolysis reactions with reported efficiencies of 85 and 74% for three- and five-cell capture, respectively. On this platform, the dynamics of immune cell cytolysis reactions using NK-92 cells as effector cells and K562 human erythroleukemia cells as target cells. The result indicated that the NK cells, which had the stronger cytolysis capabilities, overexpressed cytotoxicity and adhesion molecules (ICAM1 and B4GALT1). Dura et al. (2015) presented a trap-based method to enable pairwise-correlated multiparametric profiling of lymphocyte interactions (dynamic CD8^+^ T cells and SIINFEKL-loaded MHCII-eGFP B cells) over hundreds of pairs. The heterogeneity in early activation dynamics of CD8 T cells [OT-1 and TRP1 transnuclear (TN)] was also explored. Faley et al. (2008) demonstrated a trap-based method to delve into hematopoietic cell pairs. They investigated in the real time contact- and non-contact-based interactions between primary T cells and dendritic cells (DCs) originated from human monocytes. The same group then studied stem cells damage and chronic myeloid leukaemia (CML) (Faley et al., 2009). They investigated the responses of normal and CML CD34^+^ hematopoietic stem cell to the tyrosine kinase inhibitor, dasatinib, a drug approved for the treatment of CML. Integrating microwells and single-cell trap arrays, Jang et al. (2015) reported a platform allowing visualization of the immunological synapse (IS) in vertically “stacked” cells to investigate the interaction between single NK cell (KHYG-1 human NK cell expressing CD16) and tumor cell (K562) for the IS in a high-throughput manner. They found novel distribution of F-actin and cytolytic granules at the IS, PD1 microclusters at the NK IS, and kinetics of cytotoxicity.

The tumor microenvironment in which cancer cells, endothelial cells, and macrophages coexist could examine tumor progression (Muir et al., 2018). Frimat et al. (2011) presented a device to study tumor–stromal cell interactions. For single human epithelial cell SW480 and breast cancer cell (MCF-7)/colon carcinoma cell (HT29) co-culture, the device integrated a differential fluidic resistance trapping method with a novel cellular valving principle. The single-cell co-culture was in proximity for the formation of connexon structures and the study on contact modes of communication. Lin et al. (2013) presented a platform with hydrodynamic sieve-like traps to position cells on protein (native fibronectin) micropatterns. Moreover, Zhang et al. (2014) reported a technique called “Block-Cell-Printing” (BloC-Printing) for heterotypic breast cancer cell pairing (Figure 3B). The technique was the ability to induce cell synapse formation and directed migration. Fatsis-Kavalopoulos et al. (2019) reported a platform that enabled the organization of MIN6 β-cell and three MCF-7 cells into precise cell clusters in a flow chamber compatible under a high-resolution microscopy. They found that the changes of the concentration of cytosolic Ca^2+^ in the cancer cells were proportional to the distance from the ATP-releasing β-cell. Chen et al. (2014a) demonstrated a 3D microsystem for cancer–stromal cell interaction by co-culturing single UM-SCC-1 (head and neck squamous cell carcinoma) cell and human dermal microvascular endothelial cell. The microsystem was capable of connecting the cell culture chamber to the media exchange layer. Zhu et al. (2019) proposed a novel platform to perform cell pairing for Human Umbilical Vein Endothelial Cell (HUVEC) and cancer cell (HeLa and MCF-7) communication. On such a platform, the HUVEC could enhance HeLa cell proliferation. Moreover, He et al. (2019) demonstrated a multi-cell co-culture device to study the interactions of immune and cancer cells. Using this device, triple single-cells were performed with lymphatic endothelial cells and the human oral squamous cell carcinoma (OSCC) TW2.6 cells with the expression of WNT5B-specific shRNA (WNT5B sh4) and vector control OSCC TW2.6 pLKO-GFP cell. Tang et al. (2020) reported a microfluidic device to monitor cell–cell interaction between tumor cells (HeLa and HT 29-cell) and NIH/3T3 fibroblast cells; serpentine-like channel and traps were used for the immobilization of adjustable quantities of cells based on passive hydrodynamics. To investigate cell–cell interaction, calcein-AM transfer between multiple cells under different patterns had been quantified with local fluorescent intensity. Except for the presented application, structure traps could also be employed for other cell–cell interaction analyses, such as asymmetric trap for obstacle/particle steric interactions (Lee and Burns, 2015), pneumatic valve for parallel and dynamic monitoring processes of particle clusters interactions (Kim et al., 2017), and hydrodynamic trap for cell–cell interaction (Duchamp et al., 2019).

### Electric Field

Electric-field-based cell–cell communication studies at a single-cell level have been extensively applied (Samiei et al., 2015; Faraghat et al., 2017; Yao et al., 2019). Integrated electrodes employed dielectrophoresis (DEP) to trap single cells at the electrode interface (Wu et al., 2018). Strömberg et al. (2000) developed a single cell-pair electrofusion technique suitable for fusion between individual vesicles and proteoliposomes cells. Yin et al. (2010) presented a platform integrating microfluidic channel with DEP for single immortalized human umbilical vein cells (iHUVEC) pairing and co-culture. On the platform, single cells, cell pairs, and a small group of cell pairs were performed with DEP. The signaling output of the NF-κB (nuclear factor-k-gene binding) pathway in response to combinations of IGF1 (insulin-like growth factor 1) and TNF (tumor necrosis factor) was investigated. Activation of NF-κB with immobilized TNF and IGF1, the cell response could be abolished to different degrees by variable dose of the pathway inhibitor IκB kinase (IKK).

Bocchi et al. (2012) designed a device for homotypic cell pairs (K562 leukemia cells) integrating the inverted open microwell with DEP to regulate cell loading to the microwell and the formation of cell aggregates for cell–cell interaction studies (Figure 3C). Wu et al. (2017) developed a platform to achieve high-throughput homotypic cell pairing (more than 2,400 single cell pairs) within a 1 × 1.5 cm^2^ area by positive dielectrophoresis (p-DEP) in several minutes. Cell communication and precise cell pairing steps in cell fusion were combined. Chen et al. (2014b) developed a device that could control the co-culture microenvironment with electrolytic valving. They achieved cell–cell interaction assays between prostate cancer (PC3) cells and myoblast (C2C12) cells.

### Droplet

Microfluidics-based droplets isolate single cells and reagents in monodisperse picoliter liquid droplets (Teshima et al., 2010; Chen et al., 2018; Ahmadi et al., 2019) that can be manipulated with various cells (Joensson and Svahn, 2012). Lagus and Edd (2013) presented a device integrating droplet microfluidics with inertial microfluidics for single mating-type minus (mt–) and mating-type plus (mt+) *C. reinhardtii* cell pairs in droplets. With the reported device, about 13% of the droplets contained the correct one-to-one pairing of two separate strains of *C. reinhardtii* for long-culture analyses. Sarkar et al. (2015) put forward a droplet microfluidics-based platform to encapsulate primary T cell and DC pairs in nanoliter-volume droplets for cell–cell interaction and dynamic calcium signaling study (Figure 3D). The platform could generate and dock monodisperse nanoliter (volume 0.523 nL) droplets, capable of monitoring a thousand droplets per experiment. To assess the interaction of single T cells with dendritic cells (DCs), they reported an integrated single-cell localization, activation, and dynamic analysis platform, on which they also accessed Ag-loaded DCs activate the antitumor of CD8^+^ T cells (Sarkar et al., 2016). Chung et al. (2017) designed a promising method to enrich droplets exactly encapsulating a single particle via fluorescence or scattering-light activated sorting. With their method, two droplets, each having a remarkable particle, were precisely paired and merged in a microwell device yielding a 90% of post-sorting particles capturing rate and a total 88.1% co-encapsulation ratio. Segaliny et al. (2018) employed a droplet platform to form heterotypic cell pairs for the T cell heterogeneity study and functional TCR T cell (T cells expressing engineered T cell receptor) screening. With such a platform, single MART-1 and NY-ESO-1 engineered TCR T cell (generated by transducing Jurkat E6.1 T cells) could be activated upon recognition of target tumor cells (NY-ESO-1+K562 cell) were screened out and then monitored in the real time; it also included a system for respective clone with a 100% specificity verified by downstream single cell reverse-transcription PCR and sequencing of the TCR chains. Recently, Sullivan et al. (2020) designed a platform for immunotherapeutic applications via single-cell interactions; single CD8^+^ T cells and MDA-MB-231 breast cancer cells, and single CD8^+^ T cells and SKOV3 ovarian cancer cells were paired in 4,000 trapping sites for subsequent analyses. On this platform, two antibodies (TSR-042 and TSR-033) for the inhibition of the PD1 and LAG3 pathways were investigated for combination therapy. The results indicated that the combination of TSR-042 and TSR-033 could increase tumor cell killing at the single-cell level.

### Acoustofluidics

Another method of manipulating cells is based on acoustofluidics. Surface acoustic wave (SAW)-based methods could precisely position cells and fluids (Fakhfouri et al., 2016). Guo et al. (2015) employed a SAW-based method to regulate spatial arrangements and the distance of suspended cells to conduct quantitative investigation of the gap junctional intercellular interaction in homotypic and heterotypic groups through the visualization of fluorescent dyes transferred between cells (Figure 3E). The SAW-based method was also applied to monitor how single lymphocytes and red blood cell (RBC) were affected by the malarial parasite *Plasmodium falciparum* (Collins et al., 2015).

### Magnetic Force

Magnetic manipulation, in which magnetic beads are selectively attached to cells, is a commonly used method for single cells separation or purification in microfluidic devices (Lee et al., 2016). The magnetic field gradients can capture the magnetic beads and the attached cells from samples with large volume (Yaman et al., 2018; Pei et al., 2020). Lim et al. (2014) demonstrated scalable integrated circuits for transporting single cells along programmable trajectorys to place single lymphocytes (B cell and T cell) pairs into large arrays for the downstream analyses experiments.

### Optical Tweezers

Optical tweezers are contact-free and easily implemented in microfluidic devices for single cell research (Huang et al., 2014; Murphy et al., 2018; Zhu et al., 2018). Optical tweezers precisely control single cells to perform high-throughput analysis. Khokhlova et al. (2012) employed optical tweezers to trap and study the interaction of normal and system lupus erythematosus (SLE) red blood cells (RBCs). Quantitative determination of force parameters of normal and pathological RBC pair aggregation utilizing double-trap optical tweezers was performed. Direct measurements of aggregation speed for pairs of RBCs showed a strong difference between normal and SLE blood samples: the aggregation speed of the normal RBCs was about half of that of SLE ones. Optical tweezers were demonstrated a sensitive tool for monitoring the SLE disease and its response to drug therapies on the single cell level.

Jing et al. (2018) adopted optical tweezers to arrange a pair of human pluripotent stem cells (hPSCs) expressing negatively charged podocalyxin near each other. Using parylene-C surfaces treated with oxygen plasma, the patterned hPSCs were cultured after optical manipulations. Optical tweezers have successfully been used for unveiling protein expression information by detecting hyperosmotic stress of trapped single cells (Huang et al., 2014). As the advantages of simple microfluidic device architecture, fast cellular operation, and flexibility toward cell types and applications, optical tweezers have potential in single cell studies. In addition, other optical functions can be integrated. For example, Chen et al. (2013) employed laser-induced fusion of human embryonic stem cells (hESCs) with primary human dermal fibroblasts (HDFns) on a chip by using optical tweezers.

### Summary

The microwell method is the simplest for intercellular interaction studies at the single-cell level. Compared with the structure trap method, the main advantage of the microwell method is the high throughput capability without complex hydrodynamic channel design and accurate fluid operation. The structure trap method is one of the most common method. However, the precise control of the trap as well as the accurate fluid operation are critical to single-cell pairs. Cell clogging needs to be awared in the structure trap method when processing a large number of cells. The electric field method can capture cells with wide properties. However, proper electric fields should be applied to maintain high cell vitality. Just like the structure trap method, the droplet method needs accurate fluid operation. The merit of droplet method is the encapsulation of single cells and the processing reactions which benefits to the molecular biology research (e.g., single DNA or RNA strand study). The acoustofluidics method has the merit of causing less physiological damage to cells during the process, and it is easy to add acoustic transducers on to conventional microfluidic systems. The magnetic force method generally requires magnetic labeling of cells that may influence the biological property of the captured cells and the subsequent studies. However, the magnetic field usually covers a large area, and thus, this method is advantageous for capture specific single cells from samples of large volume. The optical tweezers method provides higher precision (down to 10 nm) than other methods. However, its applications in microfluidics for cell-based assay are still limited due to the complex operation and expensive instrumentation.

## 3D Microfluidic Systems

3D microfluidic systems deliver *in vivo*-like 3D tissue- and organ-specific microarchitectures (Sart et al., 2017; Yoshida et al., 2017). The 3D method recapitulates the cell–ECM and cell–cell interactions for 3D cell culture, biochemical signal study and drug screening (Dongeun et al., 2011; Luo et al., 2019). In this section, we mainly focus on microfluidic cell–cell communication systems by the 3D method at a single-cell level. The 3D method is classified based on the interactions of single cells with ECM or with single cells. Table 2 summarizes the 3D approaches.

**TABLE 2:** Types of microfluidic devices and their applications in 3D intercellular interaction.

**Type of device**	**ECM type**	**Application**	**Cell type**	**Reference**
Cell-ECM	Dex-TA, Dex-HA-TA, PEGDA	Long-term 3D cell culture	Human MSCs	Kamperman et al., 2017a
	TG-PEG	Osteogenic differentiation study	D1 cell	Lienemann et al., 2017
	RGD-functionalized alginate	3D cell culture	Mesenchymal stem cells (MSCs)	Utech et al., 2015
	Matrigel	Clonal acinar formation	Human prostate cell (RWPE1)	Dolega et al., 2015
	Type-I collagen	Long-term 3D cell culture	Human gastric carcinoma cell (Kato III)	Guan et al., 2014
	Type-I collagen	Long-term 3D cell culture	MCF-7 breast carcinoma cells	Håkanson et al., 2011
	Agarose gel	Long-term 3D cell culture	The breast adenocarcinoma MCF-7 human cell, human embryonic kidney (HEK) cell line 293FT	Anagnostidis et al., 2020
	Ca-alginate hydrogel	Formation of 3D tissue constructs	10 μm green and red fluorescent microspheres	Liu et al., 2017
	PAH, PSS, PEG	High-throughput sub-cellular toxicity assay	CEM cell and Hela cell	Xia et al., 2018
Cell-cell	PFPE-b-(PPG-PEGPPG)- b-PFPE	3D cell culture	Single fibroblast cell (NIH 3T3) and non-adherent T cell (EL4)	Wang et al., 2016
	PEGDA 3400	3D cell culture and drug screening	Human primary renal epithelial (HRE) cell and MCF-7 cell	Kamperman et al., 2017b
	Polyacrylamide (PAA) hydrogels	Cell microenvironment	Mammary epithelial cell (MCF10A)	Tseng et al., 2012
	Polyacrylamide (PAA) hydrogels	E-cadherin molecular tension in cell pairs	Madin–Darby canine kidney (MDCK) cells	Sim et al., 2015
	NuSil	Cell-cell coupling for human airway smooth muscle study	Primary human airway smooth muscle cells (HASMCs)	Polio et al., 2019
	Ca-alginate hydrogel	Cell-cell communication	NIH/3T3 fibroblast cells, Human bone marrow-derived mesenchymal stem cells (MSCs), human umbilical vein endothelial cells (HUVECs)	Zhang et al., 2018

### Cell–ECM

**FIGURE 4:**
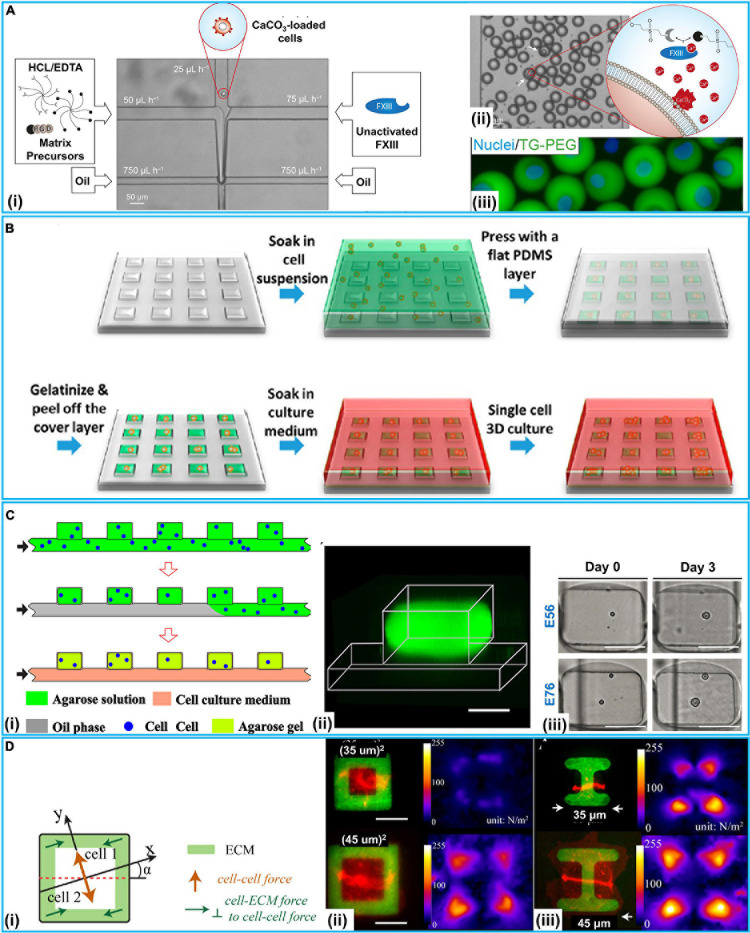
3D microfluidic intercellular interaction systems at the single-cell level. **(A)** TG-PEG-hydrogel microniches for cell–ECM communication. (i) Microfluidic chip for cell-laden TG-PEG droplet generation. (ii) Resultant TG-PEG droplets. If a cell was present in the droplet (white arrows), CaCO3 would be dissolved and Ca2+-induced activation of FXIII occurred for microniche formation. (iii) Fluorescence image of MSCs stained with Hoechst 33342 (Nuclei) encapsulated in FITC labeled TG-PEG hydrogel after retrieval from the emulsion and transfer to cell culture. Reproduced with permission from Lienemann et al. (2017), copyright 2017 The Royal Society of Chemistry. **(B)** Schematic of μCGA (micro-collagen gel array) fabrication process for 3D cell culture. Reproduced with permission from Guan et al. (2014), copyright 2014 American Chemical Society. **(C)** Microfluidic generation of hydrogel modules (HMs) for cell–cell interaction study. (i) Schematic of the generation of cell laden agarose HMs. (ii) 3D confocal fluorescence microscopy image of an individual HM formed in the well from FITC-conjugated agarose. (iii) Optical images (top view) of the cells compartmentalized in HMs for 3-day culture, scale bars: 100 μm. Reproduced with permission from Wang et al. (2016), copyright 2016 AIP Publishing. **(D)** Cell–cell and cell–ECM forces study with patterned ECM. (i) Schematic illustration of cell–cell and cell–ECM forces in cell pairs. (ii) Pairs of E-cadherin–DsRed MDCK cells spread fully on ECM squares containing collagen I and Alexa Fluor 488-labeled gelatin (green), showing a larger total force generated by the cell pair on the larger square. (iii) Pairs of E-cadherin–DsRed MDCK cells on I-shaped ECM. Reproduced with permission from Sim et al. (2015), copyright 2015 The American Society for Cell Biology.

Extracellular matrix allows cells to grow in a 3D environment with structural support. Hydrogel is a prioritized material to develop artificial ECM *in vitro* because hydrogels often consist of the materials found in the ECM *in vivo*. Kamperman et al. (2017b) developed a device for *in situ* enzymatic crosslinking of a stream of tyramine-conjugated hydrogel precursor droplets in oil through the regulated diffusing process of small crosslinker molecules. Single cell study of mesenchymal stem cells (MSCs) in crosslinked hydrogels exhibited great cell activity (>90%), metabolic activity (>70%), and multilineage differentiation capacity (>60%) for 28 days. Lienemann et al. (2017) reported a platform for selective crosslinking of cell laden pre-hydrogel droplets (TG-PEG) with synthetic microniches. As shown in Figure 4A, TG-PEG is based on two polyethylene glycol precursors that are crosslinked by the transglutaminase factor XIII (FXIII) resulting in a biocompatible nanoporous matrix. The solution of cells loaded with CaCO_3_ nanoparticles was introduced into the microfluidic chip. The TG-PEG hydrogel matrix precursor solution supplemented with hydrochloric acid (HCl)/ethylenediaminetetraacetic acid (EDTA) and the unactivated FXIII solution were also separately injected. Reagents joined in a laminar flow and were sheared by oil at a cross-junction, resulting in numerous droplets. HCl dissolved CaCO_3_ nanoparticles on the cell, leading to Ca^2+^-induced activation of FXIII and thereby on-demand crosslinking for microniche formation. EDTA could prevent background gelation occurring without a cell. Single MSCs (D1 cells) were encapsulated in TG-PEG hydrogel microniches and cultured in differentiation media for osteogenic differentiation analyses (Figure 4A). On this platform, alkaline phosphatase (ALP) expression was assessed for studying the human MSCs differentiating down the osteogenic path. To conduct single-cell–ECM communication studies with homogeneous hydrogel droplets, Utech et al. (2015) presented a method that encapsulated single MSCs in alginate hydrogels by a highly regulated manner: acetic acid was introduced in a continuing oil phase for dissociating Ca^2+^-EDTA into Ca^2+^, which released Ca^2+^ for the reaction of alginate chains. RGD-functionalized alginate was employed for encapsulating single MSCs, as RGD presented integrin binding sites for cell attachment. On this platform, single MSCs could be cultured inside the generated microenvironments for 15 days with stable encapsulation, cell growth, and proliferation. Dolega et al. (2015) demonstrated a platform for clonal acinar formation, where single cells were encapsulated in matrigel beads. In contrast to traditional bulky 3D clonal acinar formation, such a platform led to a more uniform acini population that facilitated recording the acinar developing process from the initial division to the ensuing steps.

It has long been considered that tumor cells in 3D culture can address some limitations encountered by traditional 2D monolayer cultures. For instance, 3D multicellular tumor spheroids mostly show poor sensitivity to cytotoxic drugs in contrast to cells grown on 2D substrates. Guan et al. (2014) used a micro-collagen gel array (μCGA) for 3D single-cell culture (Figure 4B). With type-I collagen, a 2 × 2 cm^2^ PDMS chip with 10,000 μCGA units was demonstrated to encapsulate considerable single tumor cells (human gastric carcinoma cells) in less than 15 min. The interacting process between tumor cell and the ECM was studied on drug responses. Håkanson et al. (2011) employed a platform to investigate the cancer cell–ECM communication. In contrast to traditional 2D protocols of cell culture and drug screening, this chip could efficiently proliferate single-cell clones. Moreover, an accurate assessment of drug effects under a lower drug concentration was presented, which did not immediately take the life of the tumor cells and avoided excess doses causing side effects. Vasileios et al. designed a device with deep neural networks accurately classifying single droplet images in real time based on the occurrence and number of micro-objects (e.g., single mammalian cells and multicellular spheroids) (Anagnostidis et al., 2020). It could identify specific objects in mixtures of objects of different types and sizes; Hek293FT cells were encapsulated in agarose gel beads for 3D cell culture with a high diversity of visual appearances. Liu et al. (2017) developed a platform integrating cell classifying and high-throughput production of 3D Ca-alginate hydrogel microstructures. The platform was produced in a smooth and efficient manner with light-addressable electro-deposition based on photoconductive material Titanyl phthalocyanine (TiOPc). This method of shaping regulated 3D gel structures did not require pre-fabrication electrodes or a 3D mold, enhancing 3D gel producing efficiency. Besides, Xia et al. (2018) reported a single-cell array for a reactive oxygen species (ROS) assay. On such an array, cell responses at the sub-cellular level, single-cell level, and population level could be overall obtained with a high throughput. As shown in Table 2, with two different cancer cells (CEM and Hela cells) and three materials {PAH [poly(allylamine hydrochloride)], PSS [poly(sodium 4-styrene sulfonate)], and PEG}, this study showed differences in responses at a single-cell level and molecular heterogeneity at sub-cellular level in considerable cells radiated.

### Cell–Cell

The cellular heterogeneity are commonly identified at the phenotypic, transcriptomic or genomic levels (Treutlein et al., 2014; Pang et al., 2015, 2016; Khoo et al., 2018; Lawson et al., 2018). Populations of multicellular constructs exhibit heterogeneities, total organizational and morphology changes have been demonstrated to simulate tumors or organs complexity (Lecault et al., 2011; Pang et al., 2019a). Microfluidics-based single-cell analysis methods have become a powerful tool to delve into the cell microenvironment, including immune cell studies. Wang et al. (2016) reported a microfluidic approach to develop cell-laden hydrogel modules (HMs) for single-cell encapsulation and culture. Single fibroblast cells (NIH/3T3) and non-adherent T cells (EL4) encapsulated in HMs achieved significant cell activity and proliferation (Figure 4C). The influences of spatial constraints and structure- and mechanics-related characteristics of HMs were examined during cell growth at a single-cell level. The interacting process between tumor cell and the extracellular matrix was studied on drug responses.

Besides, intercellular intercation regulate cell shape variations in the embryonic developing process and tissue homeostasis. Kamperman et al. (2017a) reported a modular bioink approach by the high-throughput fabrication of hydrogels (diameter 35 μm) which completely encapsulated single cells. Two distinctive major types of mammalian cell (multipotent human MSCs and bovine chondrocytes) and polyethylene glycol diacrylate (PEGDA) as a model hydrogel were tested. High-throughput microfluidics and flow cytometry-based classifying methods produced small (<40 μm) single-cell-laden hydrogels that exhibited high (>90%) encapsulation yield. Single cell PEGDA hydrogels, endothelial cells, MSCs, and proangiogenic fibrinogen macromaterial solution were used for the modular bioink. Within 1 week of culture, the angiogenic cells assembled into a CD31^+^ prevascular network throughout the construct. Tseng et al. (2012) designed a platform to study how ECM impacted the spatial organization of intercellular junctions. Fibronectin micropatterns were employed to constrain the location of mammary epithelial cell (MCF10A)-ECM adhesion. Deformations of polyacrylamide (PAA) hydrogels were used to measure the forces exerted by cell doublets on the substrate and to indirectly derive the forces they exerted on each other. By using various ECM micropatterns, they found that ECM impacted the stability of intercellular junction positioning and the magnitude of intracellular (cell–ECM) and intercellular (cell–cell) forces. Traction Force Microscopy (TFM) was used for getting images of fluorescent beads with and without cells, then the displacement field was subsequently calculated by a particle image velocimetry (PIV) program implemented as an ImageJ plugin. Specially, ECM could participate in similar morphogenetic processes. In response to heterogeneous distribution of ECM, cell doublets developed anisotropic force fields and adopted stable positions along the axis of low tension. According to this mechanism, cells tend to stabilize the position of their intercellular junctions away from the ECM.

Then, Sim et al. (2015) employed a 3D method to determine how the force balance between cell–cell and cell–ECM with varied aspect ratios and cell spread areas using pairs of Madin-Darby canine kidney (MDCK) cells (Figure 4D). By patterning ECM (collagen I/gelatin) on PAA hydrogels with micrometer resolution, various cytoskeleton strain energy states were generated. As shown in Figure 4D, E-cadherin–DsRed MDCK cell pairs were patterned on squares or I-shaped ECM structures (green). TFM was also used to test the green fluorescent beads mixed in the PAA hydrogels. Continuous peripheral ECM adhesions resulted in increased cell–cell and cell–ECM forces with a growing spread area. Specially, cell pairs maintained constant E-cadherin molecular tension and regulated total forces relative to cell spread area and shape but independent of total focal adhesion area. Recently, Polio et al. (2019) employed NuSil gel micropatterning for delving into force transmission in a two-cell ensemble of primary human airway smooth muscle cells (HASMCs). The ECM stiffness could be a switch regulating whether forces were transmitted via the cell–cell or cell–ECM contacts. Connectivity variation could significantly alter the total contractile strength of the ensemble as well.

Hydrogels having separately regulated compartments encapsulating cells would accurately regulate the path of pairing single cells. Zhang et al. (2018) employed a single-step microfluidic platform for generating monodisperse multicompartment hydrogels which could serve as a 3D matrix for pairing single cells with a high biocompatibility. Stem cells (MSCs) and niche cells (HUVECs and NIH/3T3) were entrapped in separate but adjacent hydrogel droplet compartments, capable of facilitating the study on cell–cell interactions. The method represented an essential step toward high-throughput single cell encapsulation and pairing for the study on intercellular interactions.

## Organ-On-A-Chip

The intercellular interaction is also important for organ-on-a-chip, whereas organ-on-a-chip technology can contribute to the intercellular interaction studies. In the past decade, the development of microfluidics enabled the construction of organ-on-a-chip (Zhang et al., 2018; Wu et al., 2020). For example, Huh et al. (2010) reported lung-on-a-chip where a reciprocating mechanical motion was implemented to mimic the lung alveolar motion. Ren et al. (2012) described a microfluidic device with parallel channels interconnected by micropillar arrays to mimic the capillary-myocardial tissue interface for studying hypoxia-induced myocardial injury. Fresh medium with/without oxygen consumption blocking reagent was infused into the respective adjunct channel to produce a hypoxia gradient in the middle channel to mimic the hypoperfusion/hypoxia condition during myocardial infarction. There are also other studies mimicking kidney (Wang et al., 2017), liver (Ma et al., 2016), brain (Booth and Kim, 2012), and intestine (Kim et al., 2016) by using the microfluidic chips.

However, the reported organ-on-a-chip platforms could partly mimic the organs *in vivo* (Tian et al., 2019). They simulated some physiological functions or anatomic structures, but could hardly recapitulate all the necessary environmental conditions including gas (O_2_ and CO_2_), pH, and growth factors (Wang et al., 2020). In addition to reconstruct the structures and functions *in vivo*, critical tissue interfaces, spatiotemporal cell–cell and cell–ECM interactions, and biochemical concentration gradients are desirable for further advancement of the fields of regenerative and precision medicine. Cell–cell and cell–ECM interaction at the single-cell level provide a simple and easy solution. Some tumorigenesis (e.g., breast cancer and glioma) is also closely related to single tumor stem cells and tumor microenvironment interaction (Pang et al., 2020). The single-cell intercellular interaction plays a key role in organ-on-a-chip.

## Conclusion and Outlook

Based on the advantages of microfluidics, such as low reagent consumption, precise fluid manipulation at the microliter scale and easy integration of functional components, microfluidics for the intercellular interaction study at the single-cell level has been significantly developed over the past decade. In this review, based on the way that cells interact with each other, the microfluidics-based systems are categorized into 2D and 3D methods. Considerable achievements and applications have been reported for immunology, 3D niche microenvironment, cell secretion and others. With various applied scenarios, reviewed microfluidic tools for 2D/3D cell–cell communications are listed in Table 3. Generally, 2D microfluidics-based systems are easy in operation with potential high-throughputs of cell pairs. The main advantage of 3D methods is the better controllability of cell interactions and recapitulation of the tissue architectures and extracellular microenvironments *in vivo*.

**TABLE 3:** Overview of microfluidics techniques for cell-cell communication study at a single-cell level.

**Type of device**	**Type**	**Application**	**Advantage**	**Disadvantage**
2D	Microwell	∙Immunology	∙Fusion-capable	∙Limited cell confinement in some cases
	Structure trap	∙Tumor immunology	∙High throughput	
	Electric field	∙Stem cell differentiation	∙Cell movement could be confined	∙Neglecting cell-ECM communication
	Droplet	∙Cell secretions	∙Simple to track and image	
	Acoustofluidics	∙Microenvironment	∙Cell communication	∙Easy to operate	∙Difficult to stimulate one single-cell without afflicting the other single cells
	Magnetic force	∙*P. falciparum* parasite and RBC		
	Optical tweezers			
3D	Cell-ECM	∙Stem cell culture and differentiation	∙3D cell culture	∙Complex operate
	Cell-cell	∙Tumor-initiating and tumorigenesis	∙More *in vivo*-like microenvironment	∙Low throughput
		∙Immunology	∙Communication combining cell-ECM and cell-cell	∙Difficult to image
		∙Microenvironment		
		∙Cell communication		

*The resolutions noted are nominal and may subject to change under specific operation conditions.*

Despite the exciting progress in microfluidics-based systems for intercellular interaction studies at the single-cell level, there are still challenges in its applications. First, with cell pairs, the study on cell–cell communication focuses on several important signals. However, a specific signal is difficult to be isolated precisely because of the complex signaling pathways between single cells. With several methods, e.g., cell protrusions (Zhang et al., 2019) and extracellular vesicles (Chen et al., 2019), isolating and analyzing a specific signal from the large communication information could be presented. Second, challenges remain in the studies of communication between rare cells (e.g., CTCs and tumor-initiating cells) and microenvironments (e.g., endothelial cells, macrophages, and ECM). Each single rare cell is capable of communicating with others via electrical signal transmission, soluble factors diffusion, physical contact, and ECM. The communication information is critical to tumor occurrence and metastasis. Lastly, innovative and user-friendly devices and designs need to be implemented by integrating various novel methods (e.g., digital microfluidics and 3D-printer technology) with high precision, robustness, throughput and reproducibility for research scientists in varied disciplines. Overall, with the advances in new fabrication techniques and materials, we anticipate the field would rapidly expand and be widely applied in biology and medicine.

## Author Contributions

LP, JD, ZK, LG, and X-XL wrote the manuscript. LP, XX, and S-KF revised the manuscript. All authors have read and agreed to the published version of the manuscript.

## Conflict of Interest

The authors declare that the research was conducted in the absence of any commercial or financial relationships that could be construed as a potential conflict of interest.

## Publisher’s Note

All claims expressed in this article are solely those of the authors and do not necessarily represent those of their affiliated organizations, or those of the publisher, the editors and the reviewers. Any product that may be evaluated in this article, or claim that may be made by its manufacturer, is not guaranteed or endorsed by the publisher.
